# Development and validation of a questionnaire to measure moral distress in community pharmacists

**DOI:** 10.1007/s11096-016-0413-3

**Published:** 2016-12-22

**Authors:** Jayne L. Astbury, Cathal T. Gallagher

**Affiliations:** School of Life and Medical Sciences, University of Hertfordshire, Hatfield, Herts AL10 9AB UK

**Keywords:** Community pharmacy, Moral distress, Professional ethics

## Abstract

*Background* Pharmacists work within a highly-regulated occupational sphere, and are bound by strict legal frameworks and codes of professional conduct. This regulatory environment creates the potential for moral distress to occur due to the limitations it places on acting in congruence with moral judgements. Very little research regarding this phenomenon has been undertaken in pharmacy: thus, prominent research gaps have arisen for the development of a robust tool to measure and quantify moral distress experienced in the profession. *Objective* The aim of this study was to develop an instrument to measure moral distress in community pharmacists. *Setting* Community pharmacies in the United Kingdom. *Method* This study adopted a three-phase exploratory sequential mixed-method design. Three semi-structured focus groups were then conducted to allow pharmacists to identify and explore scenarios that cause moral distress. Each of the identified scenarios were developed into a statement, which was paired with twin seven-point Likert scales to measure the frequency and intensity of the distress, respectively. Content validity, reliability, and construct validity were all tested, and the questionnaire was refined. *Main outcome measure* The successful development of the valid instrument for use in the United Kingdom. *Results* This research has led to the development of a valid and reliable instrument to measure moral distress in community pharmacists in the UK. The questionnaire has already been distributed to a large sample of community pharmacists. *Conclusion* Results from this distribution will be used to inform the formulation of coping strategies for dealing with moral distress.

## Impacts of Findings

- The recognition of moral distress is a significant barrier to well-being in community pharmacists.
- Tools could be developed to quickly assess both the frequency and intensity of distress in the population, and to determine which common scenarios precipitate it.

## Introduction

Since the term was first coined to describe some of the ethical challenges and moral conflicts inherent in the provision of nursing care [1], the definition of moral distress has undergone numerous refinements by subsequent authors [2–9]. However, the following consolidated definition, proposed by Nathaniel, encapsulates the phenomenon of moral distress in contemporary healthcare:

Moral distress is the pain affecting the mind, body or relationships that results from a patient care situation, in which the [practitioner] is aware of a moral problem, acknowledges moral responsibility and makes a moral judgement about the correct action, yet, as a result of real or perceived constraints, participates, either by act or omission, in a manner he or she perceives to be morally wrong [10].

Studies concerning moral distress in the nursing profession have identified significant negative consequences for both health of the clinician and the quality of patient care. The initial feelings of anger and outrage that are experienced during the event often develop into enduring feelings of guilt, hopelessness, loss of confidence, decreased self-esteem, exhaustion and burnout [11]. Moral distress has also been found to be associated with an exodus from the profession [12–14].

As the conceptual boundaries of moral distress have developed, so too has the research interest in the experiences of other professional groups. Although moral distress was initially delineated within nursing, the concept is relevant across other healthcare professions, as each role carries its own code of ethics, professional regulations and legal requirements to be balanced against the individual practitioner’s moral framework [15]. Subsequent studies have suggested that moral distress is relevant to and reported by various disciplines including psychiatric nurses, psychiatrists, podiatrists, psychologists, physiotherapists and respiratory therapists [16–21].

Pharmacists working in the UK operate within a highly-regulated occupational sphere, and are bound by strict legal frameworks and codes of professional conduct. This regulatory environment creates the potential for moral distress to occur due to the limitations it places on acting in congruence with moral judgements. The level of legal regulation of pharmacists compared to other healthcare professionals is marked: for example, a single error in the dispensing of medicines may be considered a criminal offence under s.64(1) of the Medicines Act 1968 [22].

Futhermore, as pharmacists expand their roles to include more clinical care, there are significantly more opportunities for ethical and moral problems to arise. Additionally, community pharmacists are generally more isolated from support networks than their hospital-based colleagues.

This research builds upon a 2015 literature review and research agenda by Astbury and co-workers for the study of moral distress in community pharmacy practice [23].

## Aim of the study

The aim of this study was to develop an instrument to measure moral distress in community pharmacists working within the UK’s National Health Service (NHS).

## Questionnaire development

### Overview

The study adopted Myers and Oetzel’s three-phase exploratory sequential mixed method design [24], as described by Creswell and Plano Clark [25]. An initial (qualitative) stage was used to explore moral distress from the perspective of practicing community pharmacists and to identify the pharmacy practice situations that they associate with experiences of moral distress. During the second stage, the qualitative findings were used to inform the development of an instrument to capture data regarding the intensity of moral distress and the frequency of its occurrence as experienced by community pharmacists. The instrument was then subjected to content validity testing before being trialed with a pilot sample in the third (quantitative) phase of the study. The results of the pilot sample were then used to carry out construct validity and reliability testing.

### Stage 1

#### Focus groups

An initial literature search was undertaken of several electronic databases including PubMed, Scopus, Web of Science and Google Scholar using combinations of the search terms “moral”, “ethical”, “distress”, “stress”, “instrument”, “scale”, and “questionnaire”. The resulting literature and existing moral distress instruments were parsed for clinical practice issues and scenarios with potential relevance to pharmacists, which were used to create an initial item pool.

Three semi-structured focus groups were then conducted to explore whether the practice scenarios highlighted in the literature review were applicable to community pharmacists within their working lives, while simultaneously providing opportunity for the participants to identify any other scenarios or issues for item development [26, 27]. The initial group session was conducted in conjunction a Royal Pharmaceutical Society (RPS) Local Practice Forum (LPF) for [REDACTED] and [REDACTED], and attracted 17 participants, 13 of which worked primarily within community pharmacy settings. A further two participants worked in each of the pharmaceutical industry and the hospital pharmacy sector, respectively. The topic guide created from the findings of the literature review was used to stimulate discussion, and participants were encouraged to raise any other issues they felt were relevant. Participants were asked to complete a demographic questionnaire as part of the registration process for the event. As there was a notable under-representation of newly-qualified and junior pharmacists in the initial group, two further focus groups were convened. The membership of these groups were made up of community-based practitioners with less than five, and ten years of post-qualification experience, respectively. These groups was drawn from alumni of the four-year *Master of Pharmacy* qualifying degree program at the University of [REDACTED], and newly-qualified pharmacists employed in its immediate vicinity. Each session lasted for approximately two hours, and each was recorded using a proprietary audio-visual recording system.

The audio recordings of the focus group sessions were transcribed verbatim and thematically coded using the broad principles of grounded theory [28]. The transcripts were read through in their entirety several times before being combined and subjected to open coding. These initial codes were then organised into categories, each of which were further divided into themes. [29] An inductive approach was applied, allowing themes to be derived from the data using open coding, grouping and categorising. This enabled abstraction and conceptual mapping to create a resonant description of the phenomenon [30]. The category content was re-examined and compared at various stages throughout the analysis, and categories felt to capture the same entity within the data were merged and reconfigured. Coding was carried out using test–retest methodology with a 1 month coding interval [31].

**Fig. 1:**
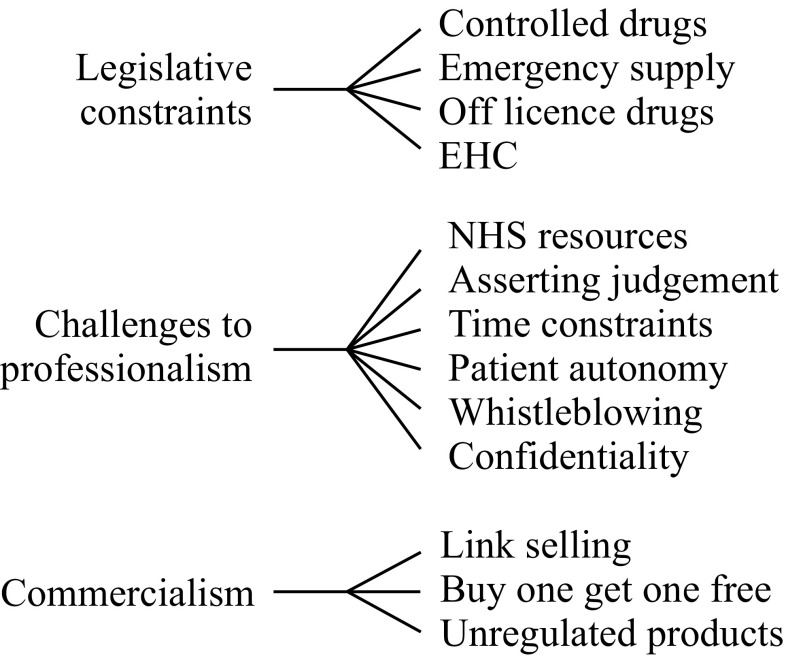
Categories (*left*) and themes (*right*) emerging from initial thematic analysis

Four categories relating to moral distress were identified, namely: legislative constraints; commercial pressures; challenges to professionalism; and risk taking & resilience. Fifteen individual themes, including emergency hormonal contraception (EHC), whistleblowing, and patient confidentiality were identified within the four categories; of which thirteen themes in three groups related directly to causes of moral distress (Fig. 1).

#### Legislative constraints

It was in the category of legislative constraints that the potential for moral distress was most immediately obvious: in the scenarios described by participants, acting in accordance with their respective consciences would have resulted in a breach of statutory law. The scenarios that appeared to be most strongly associated with the experience of moral distress concerned situations in which the pharmacists felt unable to lawfully dispense controlled drugs despite their belief that to do so would be in the patient’s best interests. The Misuse of Drugs Regulations 2001 place unambiguous procedural requirements on pharmacists regarding the storage, supply, and use of medicines that are classified as controlled drugs by the Misuse of Drugs Act 1971 (as amended) [32, 33].

Contravention of the regulations constitutes a criminal offence, and may additionally constitute an impairment of the pharmacist’s fitness to practise under the General Pharmaceutical Council (Fitness to Practise and Disqualification etc.) Rules 2010 [34]. A finding of impairment by the General Pharmaceutical Council’s (GPhC) Fitness to Practise Committee is associated with sanctions ranging from a warning to removal of the pharmacist from the Register of Pharmacists. In the focus groups, the pharmacists described situations in which they felt confident that the request made by the patient was legitimate, but that the required procedural aspects of dispensing could not be complied with due to absent or incorrectly written prescriptions presented at a time when sourcing a replacement was logistically difficult (such as on a Sunday or outside normal business hours). In these situations the perceived needs of the patient conflicted with the professional requirement to act within legislative guidelines. When coupled with an acute awareness of the potential personal consequences of acting outside of the regulations, the potential for moral distress is clear.

Practice scenarios that the pharmacists shared often involved the supply of methadone as part of pharmacological withdrawal treatment for patients addicted to heroin. For pharmacists working consistently from the same pharmacy the frequent and regular contact with patients using the methadone service created a heightened sense of professional involvement in, and engagement with, the individual’s treatment plan and wellbeing. The pharmacists spoke of an acute awareness of the possible consequences for the individual patient of not supplying, and their distress at being unable to do what they felt was in the patient’s best interests:

At the end of the day, you’ve got someone who is a family member, that’s going to be somebody’s mother, somebody’s father, somebody’s husband, somebody’s wife. I am never comfortable with not dispensing [methadone]. If I don’t supply this guy, he’s going to start using [heroin]. I want to keep the guy clean, if he starts using again he goes backwards, and that’s no use to anyone.

Similar legislative and professional constraints are associated with the supply of EHC and of the supply in an emergency of prescription-only medicines (POMs) without a prescription, and with the requirement to breach patient confidentiality under legislation unrelated to the provision of healthcare.

#### Commercialism

In May 2001, the exemption from the general ban on resale price maintenance enjoyed by proprietary non-prescription medicines was removed. The Proprietary Association of Great Britain (PAGB) withdrew their opposition to this removal following an indication by the Restrictive Practices Court that it was unsympathetic to the points they were making [35]. Since that time, pharmacies have been permitted to offer promotions on the sale of medicines that were previously prohibited under the Restrictive Practices Court (Resale Prices) Rules 1976 [36]. This has, in the opinion of focus group members, lead to a degree of commercialisation that conflicts with the core professional values of pharmacy. A number of the pharmacists described feeling pressurised to generate and influence purchases that were not necessarily required or advised. They felt compelled to promote and facilitate commercial incentives even when they conflicted with their professional opinion regarding optimal use of medicines.

Feeling compelled to generate additional sales through the use of “three-for-two” offers and linked-selling strategies was cited as a source of moral distress by a number of participants. In addition, pressure from employers to promote and sell unregulated products such as e-cigarettes, homeopathic products and slimming aids was also cited as a cause of moral distress by some.

I feel I’m expected – due to my professional standing – to promote the sale or supply of products that have not been proven effective, or that have been proven ineffective, so I don’t like selling homeopathic products … I feel we shouldn’t be selling them in pharmacies because they are not medicines, they work contrary to what we are told.

The perceived pressure to prioritise sales targets over customer needs was echoed throughout the focus groups.

#### Challenges to professionalism

Six themes in total were identified under the category of “challenges to professionalism”, namely: NHS resources; asserting clinical judgement; time constraints; patient autonomy; whistleblowing; and confidentiality. Each of these themes arose from situations in which the pharmacist was required to “speak up” against a decision that another party was trying to impose upon them. Unlike the other categories, in which there were discrete penalties or sanctions for acting with their conscience, the decision-making process here tends to be affected by the fear less tangible consequences, such as the erosion working relationships or the loss of autonomy. The major hurdle that must be overcome is the assertion of professional judgement in the face of others who may disagree with it.

For example, one participant was particularly concerned that some patients’ habitual failure to collect expensive made-to-order medicines in a timely manner constituted a waste of NHS resources and public money, but felt unable to challenge this behaviour:

There are also patients that need some creams, or some ‘specials’ made up for them and they don’t come to collect them, and I feel so bad because the creams they expire [quickly], sometimes in only a few days. They cost a lot. It happens a lot.

One theme consistently raised in this category was that of the use by savvy patients of medicines outside their licensed indications.

Participants raised a number of situations concerning requests for medicines to be used outside of their officially-licensed indications. Specifically, the pharmacists highlighted situations in which they suspected that medicines were being sourced for reasons other than those described by the patient. A focus group participant provided the following example in which a customer made repeated visits to the pharmacy to request a specific antihistamine which the participant suspected was being used as a sedative for a child:

There is so much going on in your head because, you know, they are there asking for two or three boxes of [sedative antihistamine], saying, “It’s for me and my husband, for allergies.” But I know she has also got an 8-year-old and you know that this is just a story they tell to get it.

Acquiescing to the customer’s requests for medicines for uses outside of licensed indications created feelings of unease and conflict due to concerns that this constituted poor professional judgement and practice, and facilitated the misuse of medicines.

#### Risk taking and resilience

Not all of the themes identified involved scenarios that could result in moral distress. The final category described instances where pharmacists deviated from the rules governing from their profession in order to avoid moral distress. Kälvemark and co-workers also reported instances in which pharmacists ignored legal and professional requirements in order to act in congruence with what they felt was morally right [37]. Such avoidance strategies straddled each of other categories and their themes. For example, rather than suffer the moral distress associated with legally declining to supply a CD in an emergency:

I would dispense it, and I’ve done that before, and I’d do it again. I’m sure it’s illegal and I accept that, but at the end of the day I have a duty of care to that patient.

The most frequently cited motivating value for deciding to act against regulatory requirements was a concern for the patient’s welfare.

Newly-qualified pharmacists described feeling particularly vulnerable to experiences of moral distress whilst navigating the transition from being a student to qualified pharmacist. This period of role adaptation may be associated with a sense of generalised anxiety regarding the marked increase in levels of professional responsibility and accountability, which, in turn, makes these “adolescent” professionals more likely to experience moral distress as they strive to adhere to legislative and procedural requirements. Adolescent professionals may also face an elevated risk of moral distress due to the additional challenge of asserting their professional judgement with senior colleagues [38, 39].

### Stage 2

#### Item generation

**Fig. 2:**
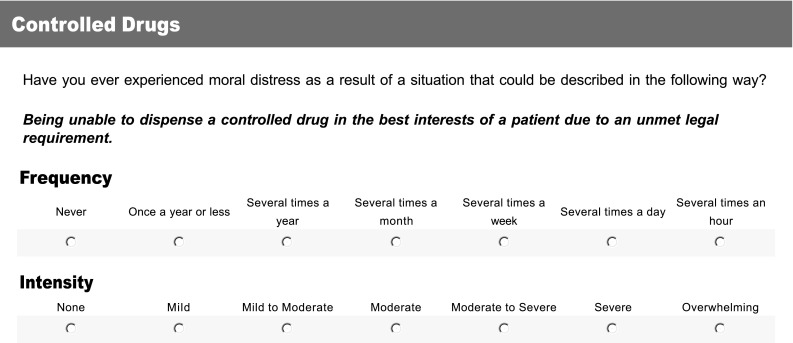
Item 1 (Controlled Drugs) as it appears on the online pilot survey for the questionnaire. Matching 7-point Likert scales for each of the two dimensions in which moral distress is to be measured are included for each of the 13 items

Each of the 13 themes relating to practice scenarios were developed into a statement that described a practice situation that could generate moral distress. A seven point Likert scale was chosen for this instrument, with each item being rated for both intensity and frequency. Each item asked the same question, “Have you ever experienced moral distress as a result of a situation that could be described in the following way?”, before going on to describe a practice scenario in a single statement (Fig. 2). For example, the scenario for EHC was described as follows: “Dispensing emergency hormonal contraception though this conflicts with my moral beliefs.”

#### Content validity

In order to explore the content validity of the instrument, the item pool and questionnaire format were submitted for review to a panel of 12 academics working in the Department of Pharmacy at the University of Hertfordshire. Reviewers were selected as described by Grant and Davis [40], taking in account their academic interests and professional experience of community pharmacy practice. They were asked to consider and comment on the clarity of the introductory text and general layout of the questionnaire. They were also required to evaluate the relevance of each item to the concept of moral distress, together with the clarity and conciseness of each item. Suggestions for additional item domains were also encouraged to ensure that the item set reflected the construct of moral distress in its totality. Reviewers rated the relevance of each item on a four point Likert Scale (1 = not relevant; 2 = somewhat relevant; 3 = quite relevant; 4 = highly relevant). This data was then used to compute item-level (I-CVI) and scale-level (S-CVI) content validity index scores (Table 1) [41]. Polit and Beck recommend that I-CVI values of above 0.9 and S-CVI values of 0.78 be considered indicative of excellent content validity [41]. Only item 13 (unregulated products) fell below the cut-off: however, it was decided to retain this item in the initial piloting due to the strong emphasis that was placed on this issue in the focus groups.

**Table 1:** Item-level (I-CVI) and scale-level (S-CVI) content validity index scores for the 13-item moral distress questionnaire

Item	Description	I-CVI
1	Supply of controlled drugs in the best interest of a patient when legal requirements are unmet	1.00
2	Wasting NHS resources to elicit patient compliance	1.00
3	Actively challenging prescribers regarding prescriptions that contained medicines or doses thought to be inappropriate	1.00
4	Feeling unable to provide an adequate level of service due to time constraints	1.00
5	Professional judgement conflicts with the preferences and wishes of the customer	1.00
6	Commercial values and a pressure to link sell to generate additional sales	0.91
7	Emergency supply of POMs when procedural requirements are unmet	1.00
8	Request from patients for medication for use outside of their licenced indications	0.91
9	Supply of emergency hormonal contraception in conflict with religious or moral beliefs	1.00
10	Professional requirement to engage in whistleblowing though this may be to the detriment of one’s career	1.00
11	Compulsion to release confidential patient data under non-healthcare-related legislation	1.00
12	Commercial incentives that are in opposition to best clinical practice	0.91
13	The sale of unregulated or unproven products	0.75
	S-CVI	0.96

### Stage 3

#### Piloting

A hyperlink and invitation to pilot the questionnaire was emailed to members of two LPFs and the National Institute for Health Research (NIHR) Clinical Research Network (CRN) Eastern region. The pilot was closed when a sample of 50 community pharmacists had completed the self-administered online survey. All of the respondents were working in a community pharmacy or undertook regular additional work in a community pharmacy setting (Table 2). An additional response box was added at the end of the pilot questionnaire inviting comments regarding the content and structure of the questionnaire. Feedback indicated that one item (unregulated products) lacked clarity and required rewording. Only one participant did not complete every aspect of the questionnaire, indicating that the scenarios held relevance for the respondents.

**Table 2:** Demographic data for the 50 participants in the pilot sample

Pharmacist	n = 50
Gender	
Female	29
Male	17
Missing	4
Age (years)	
Under 25	–
26–35	6
36–45	8
46–55	15
56–65	12
65+	5
Missing	4
Post-qualification experience (years)	
Less than 5	3
6–10	4
11–15	6
16–20	7
20+	26
Missing	4
Primary area of pharmacy practice	
Community owner	2
Community employee	19
Community locum	18
Primary care	1
Hospital	4
Pharmaceutical industry	1
Academia	1
Missing	4
Regular additional area of pharmacy practice	
Community	6
Primary care	–
Pharmaceutical industry	–
Hospital	2
Academia	1
Missing	41

#### Reliability

Both the frequency (α = 0.801; n = 50) and intensity (α = 0.816; n = 50) subscales were found to have a good level of internal consistency. Inspection of the item total correlations revealed that only the removal of item 11 (confidentiality) would have created an increase in either value of α: however, this increase was so small as to be considered negligible (0.004 and 0.005 for frequency and intensity, respectively).

#### Principal component analysis

The sample used for the pilot study was insufficiently large to allow either a meaningful principal component analysis (PCA) or Spearman’s rho to be conducted: however, returns from a larger probability sample (n = 1340), which was subsequently distributed, did allow for construct validity and reliability calculations to be carried out.

The 13 frequency subscale items were subjected to principal component analysis (PCA) using varimax rotation following a favourable assessment of sampling adequacy using the Kaiser–Meyer–Olkin (KMO) measure (KMO = 0.892) [42]. Bartlett’s test supported the factorability of the correlation matrix (χ^2^(78) = 1869.444, *p* < 0.001) [43]. Criterion for factor loadings was set at 0.30 or greater. PCA revealed the presence of two categories with eigenvalues exceeding 1.0, explaining a total 43.28% of the variance: the first component accounting for 34.75%; the second accounting for 8.50%. The majority of items (n = 12) loaded on their respective categories at above 0.50. There was a moderate positive correlation between the two categories (r = 0.399). Inspection of the scree plot revealed a clear point of inflection after the first component, indicating that only the first component should be retained. Parallel analysis provided further support for the retention of a single component [44]. The first component returned a criterion eigenvalue of 1.252 against an actual value of 4.518 from the PCA, while the second component returned a criterion value (1.186) that was significantly higher than that derived from PCA (1.105).

For the 13 intensity subscale items a KMO value of 0.905 was returned. Again, Bartlett’s test returned a favourable result (χ^2^(78) = 1995.501, *p* < 0.001), and the criterion for factor loadings was set at 0.30 or greater. Two categories accounted for 37.44 and 8.59% of total variance, respectively. Eleven items loaded on their respective categories at above 0.50. As before, there was a moderate positive correlation between the two (r = 0.422). Inspection of the scree plot again revealed a clear point of inflection after the first component indicating that only one should be retained. This was further supported by parallel analysis, in which the criterion eigenvalue (1.252) was less than the derived value (4.867) for the first component only.

The single-category structure was found to be comprised of the same item variables for both frequency and intensity subscales. The item clusters on each category indicated that the original themes were highly correlated, and could be reduced to a single category.

#### Construct validity

Construct validity was explored through correlation of individual item subscale scores with the summated score of a truncated version of the Ethical Environment Questionnaire (EEQ) [45], which was appended to the moral distress questionnaire for optional completion. The Cronbach α of this abbreviated scale was 0.79. Moral distress has been previously found to be negatively correlated with perceptions of ethical environment in studies concerning nurses and physicians, with high levels of moral distress being associated with low perceptions of ethical environment [13, 14, 46]. The relationship between individual intensity and frequency scores and the EEQ score was explored using Spearman’s rank *correlation* coefficient (n = 529). A statistically significant negative correlation was observed between the two variables for all but one item on both the frequency and intensity subscales, with low levels of perceived ethical environment being associated high levels of moral distress, confirming the predicted relationship.

## Discussion

This research has led to the development of a valid and reliable instrument to measure moral distress in community pharmacists in the UK. The questionnaire has already been distributed to a large sample of community pharmacists. An e-mail inviting pharmacists to participate was successfully delivered to the mailboxes of 20,433 recipients. 50.7% (10,360) of recipients opened the e-mail. This compares to an average of 37.8% (equivalent to 7724 recipients opening the e-mail) for distributions on this list, and an industry average in the non-profit sector of 20.3% (4148). 1618 (15.6%) of those recipients who opened the e-mail clicked through to the survey. The expected response, based on industry averages, would be 450 (4.3%). A total of 1340 pharmacists completed the survey following a reminder.

In developing a questionnaire of this type, it is important to consider how the collected data will be treated. There are two approaches to the interpretation of questionnaires of this type: individual recording and cumulative scoring. Although both methods appear throughout the literature, we contend that the latter is often invalid, due to the nature of the points on Likert-type scales.

Likert scales are presented as linear scales with the equidistant differences between interval points: however the differences in attitudinal intensity between the intervals cannot be precisely quantified [47]. On the intensity scale, the separation between *mild to moderate* and *moderate to severe* intensity cannot be assumed to be the same; while on the frequency subscales, the interval points refer to easily recognised measures of time that do not have a linear relationship with each other, but that better reflect how people recall the recurrence of events. Cumulative scoring, although common in instruments of this kind [48], is premised on a known and quantifiable relationship between intervals. In the former scale, there is no such relationship. Furthermore, even on the frequency scale, where such a relationship does exist, two identical cumulative scores can be derived from significantly different sets of sub-scores.

## Conclusion

For these reasons, including an interpretation of individual item responses has been suggested to provide more a more meaningful reading of the data [49]. It is intended that each item of this questionnaire be reported separately, and that items measuring the moral distress associated with different themes in the same category be subsequently compared with a view to determining which aspects of practice cause the greatest degree of moral stress.

Factors affecting scenarios occurring with lower frequencies will be examined to determine to determine if these may be applied to scenarios with high recurrence rates, with a view to reducing these rates. For example, if the GPhC’s guidance with regard to one the scenario generating moral distress is essentially pragmatic, while another is paternalistic or deontological, an examination of the consistency of such guidance would be warranted. Similarly, those scenarios scoring lower for intensity can be compared, and common factors identified, with a view to developing coping strategies for higher-scoring scenarios.

Age, experience, gender, and religious background have all been shown to have an effect on susceptibility to moral distress in other healthcare professions [6, 7, 11, 18, 20, 21, 50], and will be examined in detail. It is hoped that the results of this large-scale survey will help in the development of strategies to reduce both the frequency and intensity of moral distress in the pharmacy profession.
